# Secular Changes of Adiposity and Motor Development in Czech Preschool Children: Lifestyle Changes in Fifty-Five Year Retrospective Study

**DOI:** 10.1155/2015/823841

**Published:** 2015-08-25

**Authors:** Petr Sedlak, Jana Pařízková, Robert Daniš, Hana Dvořáková, Jana Vignerová

**Affiliations:** ^1^Department of Anthropology and Human Genetics, Faculty of Science, Charles University in Prague, Vinicna 7, 128 44 Prague, Czech Republic; ^2^Obesity Management Centre, Institute of Endocrinology, Narodni Trida 8, 116 94 Prague, Czech Republic; ^3^Faculty of Education, Charles University in Prague, M.D. Rettigove 4, 116 39 Prague, Czech Republic

## Abstract

Secular trends of adiposity and motor development in preschool children since the fifties of the last century up to the beginning of this millennium were analyzed so as to reveal possible changes due to continuously differentiating lifestyle. In preschool children (*n* = 3678) height, weight, skinfold thickness over triceps, subscapular, and suprailiac were measured by Harpenden caliper in 1957, 1977, 1980, 1985, 1990, and 2012. Simultaneously, motor performance was tested by evaluating the achievements in broad jump and throwing a ball, as a marker of adaptation to changing level of physical activity, free games, and exercise. Along the period of five decades the values of skinfold thickness increased significantly until 2012, mainly on the trunk. Simultaneously, the level of motor performance significantly decreased. Modifications of the way of life during the mentioned five decades characterized by sedentarism and inadequate food intake as related to energy output influenced negatively both adiposity and motor performance already in preschool children. Mostly increased deposition of fat on the trunk which is considered as a marker of possible development of metabolic syndrome was apparent already in preschool age, indicating the importance of early intervention concerning also physical activity and availability for exercise since early life.

## 1. Introduction

Secular changes in the way of life, which includes food intake not corresponding to energy expenditure due to considerable reduction of physical activity and work load, have resulted in a number of morphological, functional, and motor consequences which have had also a significant impact on health [1–6]. This has concerned all age categories, and especially growing subjects. Secular comparisons of school children and adolescents revealed marked increase of body fat evaluated from skinfolds [7, 8]; along with that functional capacity has deteriorated, as indicated by the decrease of aerobic power, speed, strength, and so forth, [9, 10]. As shown, for example, by Cattaneo et al. [11] and Pařízková et al. [12, 13], the increase of adiposity characterized by skinfold thickness was found already in preschool age, due also to decreasing age of adiposity rebound (AR) [14, 15]. All these changes concerning increasing adiposity have been accompanied by the worsening of motor development as a marker of the adaptation to reduced physical activity already in young children, especially when energy intake and composition of nutrition have not corresponded adequately.

When comparing the achievements of, for example, jump from the spot or ball throw by both hands, the results assessed at the beginning of this millenium and those, for example, in the seventies of the last century, recent results were significantly worse [12, 13, 16]. Deterioration of motor abilities concerns especially those which require certain experience and adaptation to adequately increasd level of physical activities mostly outside, or under special conditions. For that reason this deterioration can be considered as a marker of sedentarism and reduced participation in exercise, which used to be considerably higher decades of years ago when children had better opportunity for spontaneous games in safe playgrounds, parks, gardens, and so forth [12, 17, 18]. This is mostly not available at present [3, 6, 19], especially in larger urban agglomerations, where safety cannot be guaranteed (traffic, criminality concerning children, etc.). Unfortunately, this is reflected by both inadequate body composition—increase of adiposity (often even without increased body mass index—BMI), simultaneously with the lower level of motor development [12, 13].

This negative effect of changed lifestyle has concerned especially the critical period of development, that is, adiposity rebound (AR) when, for example, BMI [14], directly assessed adiposity (skinfolds), and level of spontaneous physical activity have been changing in a decisive manner [20]. The age of AR has been more recently decreasing due to increasing adiposity at earlier age which coincides with increasing prevalence of obesity [15]. Special attention should be therefore paid to this developmental period, with regard to nutrition and physical activity regimes which can have due to varying sensitivity to the environmental factors various consequences from the point of view of not only immediate but also delayed affects which could appear only later in life [12, 21].

*Aim of this study* was to evaluate in greater detail secular changes of adiposity in preschool age, with regard to various time periods (1957, 1977, 1980, 1985, 1990, and 2012) and to determine more exactly when the change was most marked and critical. The changes of the distribution, with special regard to trunk accumulation of fat, which is considered to be linked with possible development of metabolic syndrome later, were also followed [22, 23]. Special attention was also focused on mentioned changes of adiposity as related to the changes of adiposity rebound (AR), evaluated at the same periods of time from BMI curves.

## 2. Material and Methods

The Institutional Research Ethics Committee at the Institute of Endocrinology in Prague, Czech Republic, approved the study. Written informed consent was obtained from parents of all children participating in the study.

### 2.1. Participants

Data from 6 samples of 5- to 6-year-old children followed up in 1957 and 2012 were used for the comparison of morphological parameters and markers of adiposity (Table 1). Kindergarten children from different regions of the Czech Republic, Prague and central Bohemia (1957 [24], 1977 [25], 1980 [26], 1985 [27], and 1990 [28]; southern Bohemia (2012)) were included in our study.

Testing of motor abilities was a part of experimental measurements conducted in 1977 and in 2012 [25, 29]. The comparison of the age of adiposity rebound (AR) was based on the measurements in the framework of national anthropological research studies in children and adolescents (NAS), which have been since also used as national somatic development standards of Czech population. The NAS has been conducted at 10-year intervals, starting in 1951 [30]. Last NAS was conducted in 2001 [31]. For that reason we used for the comparison NAS data from 1951 (*n* = 126,082; 62,742 boys and 63,340 girls), 1981 (*n* = 87,316; 42,832 boys and 44,484 girls), 1991 (*n* = 70,299; 34,640 boys and 35,659 girls), and NAS 2001 (*n* = 59,082; 28,146 boys and 30,963 girls). Data from 1961 and 1971 were not available. Analysis of BMI data aimed for age determination AR was conducted in children aged 2,5–18 years.

### 2.2. Anthropometric Measures and Motor Ability Assessment

Children were of middle class background; they were always followed up in their underwear during the morning session in their kindergartens, after enough sleep and more than one hour after breakfast. Their health status was always absolutely normal, and children with even minor indisposition were not measured.

Height and weight were measured for each child. Anthropometry was conducted in accordance with the guidelines provided by Tanner and Whitehouse [32]. Body mass index (BMI) was calculated as body weight (in kilograms)/body height^2^ (in meters). For the construction of BMI percentile curves for Czech population the LMS method was used [33], based on the Box-Cox power transformation. AR was defined as the time position of the local minimum on the fitted smooth BMI curve.

The skinfold thickness was measured by Harpenden caliper (in millimeters) in suprailiac and subscapular region and over the triceps [25]. Monitored parameters are shown in Table 2.

*Motor performance* was tested by the evaluation of the results of broad jump, characterizing coordination and strength of lower extremities. Testing was conducted mostly outdoors in a playground or on a pathway in a park, not on a concrete or pavement. These tests represent simple activities usually included in children's games in kindergartens or during family activites; however, at the beginning of testing children were always instructed and demonstrated exactly how to perform.

The standing broad jump is a test for the evaluation of explosive strength of the lower extremities and also of coordination and skill. The experimental worker demonstrates the jump and instructs the child, “sway your arms and jump as far as possible!” Two attempts are recorded in cm, from the toes to the last foot mark. Throwing a ball (tennis) characterizes the explosive strength of the upper extremities, coordination, and skill. The child stands on the starting line and throws the ball with the upper arch; two attempts are conducted and the results of the better one—as in previous test—is considered [18]. These two motor abilities that were tested are more dependent on previous experience and adaptation. All methods including skinfold measurements were verified and used repeatedly in previous studies always mostly conducted directly or with participation of one of the coauthors since 1957 [12, 13, 16–18, 24, 34] and were well accepted as a game by all children.

### 2.3. Statistical Analysis

The results of motor tests (broad jump from the spot and throwing a ball) and of skinfold thickness were analysed using STATISTICA software v. 9 (StatSoft, Czech Republic). To get informations on a long-term trend of changes, skinfold and motor performance data assessed in our studies were analyzed. Before statistical testing, the data were transformed to follow a normal distribution. First, we tested the skinfold data from the studies conducted in the years 1957, 1977, 1980, 1985, 1990, and 2012 using three-way ANOVA. The effect of following factors was considered: year of study, age, and gender. Interactions of these factors have also been evaluated. False significance has been handled with Bonferroni correction. Next, the data on motor performance (from the years 1977 and 2012) were analyzed using two-way ANOVA, taking into account the age and gender as factors.

## 3. Results

**Figure 1:**
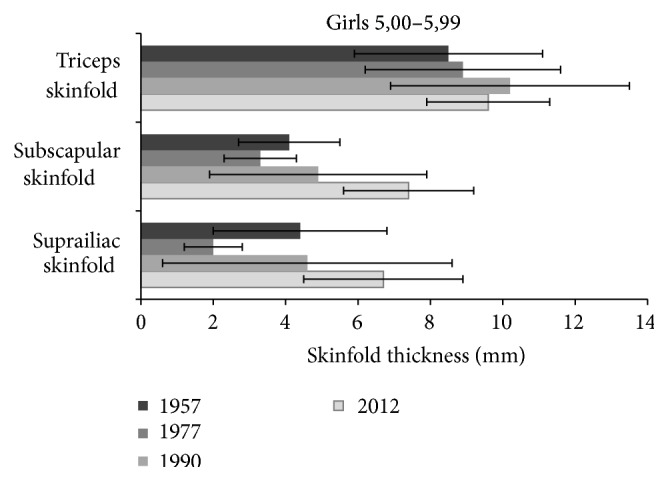
Changes of skinfold thickness (triceps, subscapular, and suprailiac) in 5-year girls from the studies of 1957 up to 2012.

As shown in Figure 1, in 5-year-old girls there was an apparent increase of skinfold thickness over the period from 1977 to 2012. Skinfolds on the trunk, subscapular, and suprailiac increased most markedly.

Significance of differences in triceps skinfold thickness of girls between 1957 and 1990 was *P* < 0.01, for 1957 and 2012, 1977 and 1990, and 2012 (*P* < 0.05). With regard to subscapular skinfold, values from 2012 and all other measurements were highly significant (*P* < 0.001), and in comparison with 1977 and 1990 they were *P* < 0.05. For suprailiac skinfold all differences were highly significant (*P* < 0.001).

**Figure 2:**
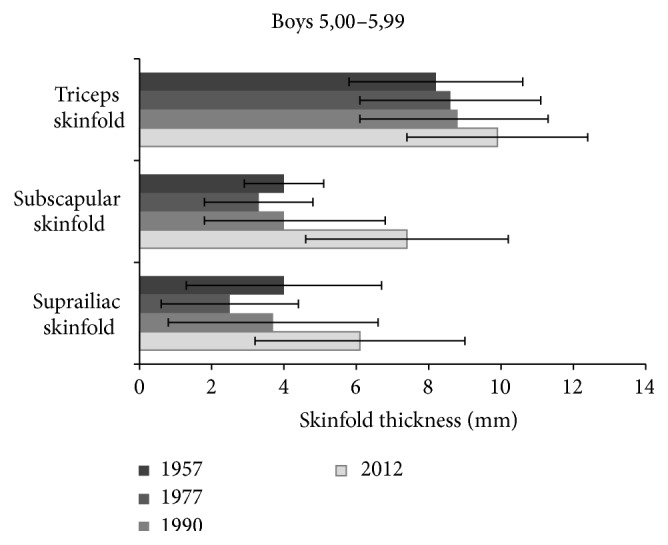
Changes of skinfold thickness (triceps, subscapular, and suprailiac) in 5-year boys from the studies of 1957 up to 2012.

Triceps skinfold thickness in boys increased significantly between the years 1957 and 1977, 1990, 2012 (*P* < 0.001), 2012 and 1977, 1990 (*P* < 0.05), for subscapular skinfold between the years 1957 and 1977, 1990, 2012 (*P* < 0.001). For suprailiac skinfold difference was significant when comparing 1957, 1977 and 2012 (*P* < 0.001), 1957, 1977, and 1990 (*P* < 0.05) (Figure 2).

**Figure 3:**
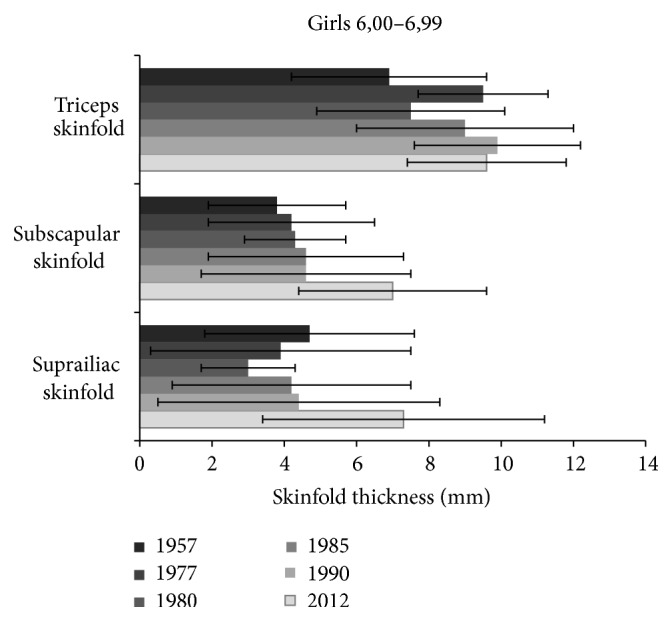
Changes of skinfold thickness (triceps, subscapular, and suprailiac) in 6-year girls from the studies of 1957 up to 2012.

**Figure 4:**
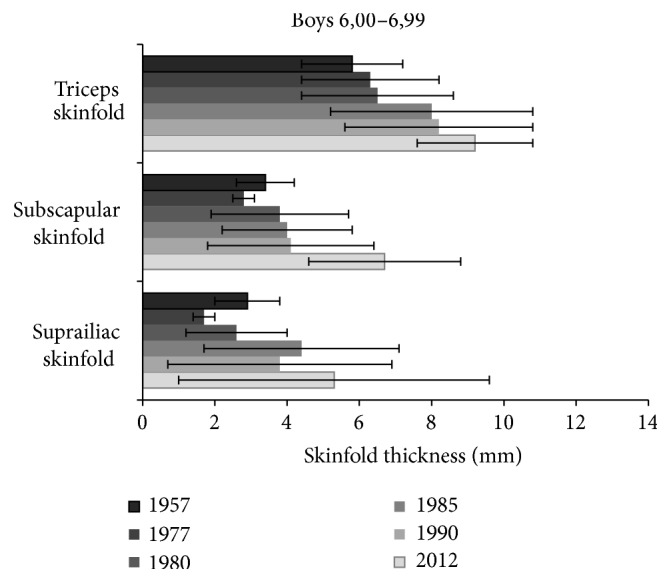
Changes of skinfold thickness (triceps, subscapular, and suprailiac) in 6-year boys from the studies of 1957 up to 2012.

In 6-year-old children the increase between the year 1957 and 1990 was not very apparent (Figures 3 and 4). Marked change, however, appeared at the occasion of most recent measurements (in 2012), when skinfold thickness on the trunk in both boys and girls increased most significantly as compared to all other previous measurements.

Further marked changes were found in girls with regard to triceps skinfold measured in 1957, 1980, and 1977, 1985, 1990 (*P* < 0.001), 1985 and 1990 (*P* < 0.05), suprailiac skinfold between the years 1980 and 1957, 1977, 1985, and 1990 (*P* < 0.05). In boys significant differences in triceps skinfold were assessed in 1957, 1977, 1980 and 1985, 1990 (*P* < 0.01), in subscapular skinfold between years 1977 and 1980, 1985, 1990 (*P* < 0.05), in suprailiac skinfold between 1977 and 1957, 1980 (*P* < 0.05), 1977 and 1985, 1990 (*P* < 0.001), 1980 and 1985, 1990 (*P* < 0.01).  Table 3 shows an overview on statistical significance of differences among the results of skinfold thickness assessed in the individual studies, as related to recent data from the year 2012.

**Figure 5:**
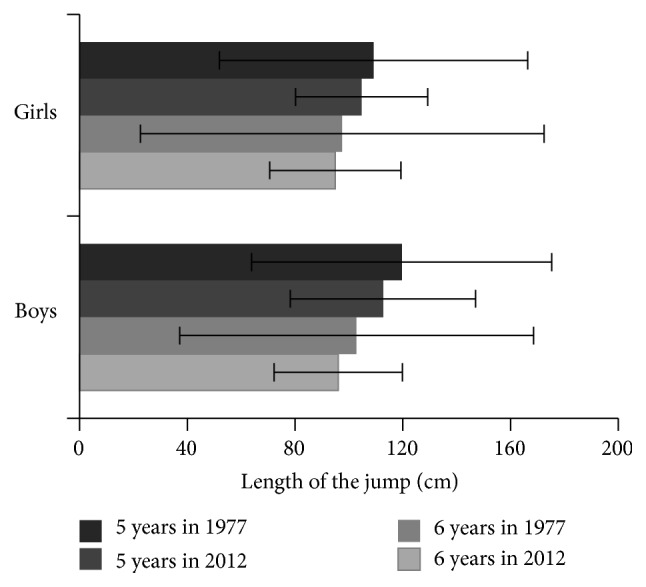
Changes of the performance in broad jump from the year of 1977 up to 2012 in preschool children in two age categories. The differences were significant in boys on the level of *P* < 0.05 in 5- and 6-year-olds; in girls, the differences were significant on the level of *P* < 0.05 only in 5-year-olds.

**Figure 6:**
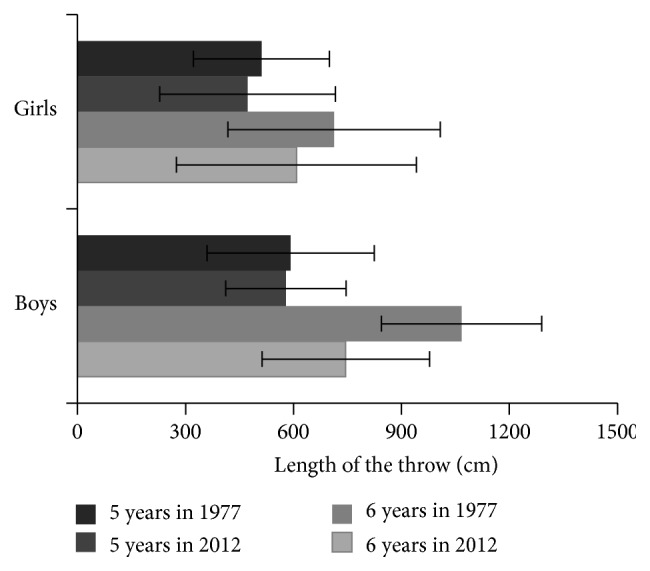
Changes of the performance in ball throw from the year of 1977 up to 2012 in preschool children in two age categories. In 6-year boys and girls, the differences were significant on the level of *P* < 0.001, in 5-year-old girls *P* < 0.05.

Development of motor skills in present preschool children is shown in Figures 5 and 6. Changes in body composition were found to be associated with the changes in motor development of preschool children. In both genders, tested aspects of motor development, explosive strength of lower extremities (standing broad jump) and manipulative skills of upper extremities (throwing the ball), were significantly worse most recently, as compared to the results assessed in their peers in 70 years of 20th century. Mostly significant drop was found especially in the sample of contemporary 6-year boys (*P* < 0.001).

**Figure 7:**
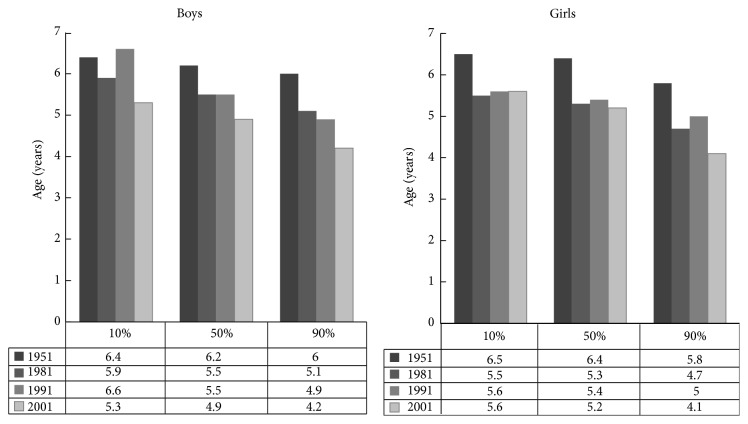
Age of adiposity rebound between the years 1957–2001 in Czech girls and boys: 10th, 50th, and 90th percentile of BMI.

As shown in Figure 7 the age of AR decreased significantly in all BMI categories. Most marked decrease of AR age occurred in children with highest weight.

## 4. Discussion

Relative stability of BMI which basically relates weight to height indicates that both of these parameters of somatic development have increased correspondingly during the period of our measurements [12, 13]. This is partly in contrast with skinfold thickness measurements, which disclose significant changes in body composition—increase of adiposity and possibly of slightly reduced development of other bodily tissues as muscles due to insufficient stimulation by physical activity and exercise [18, 35]. As total amount of total body fat and distribution of subcutaneous fat and their interrelationships in preschool age are not the same as in school age and adolescents, it was preferred to use for secular comparisons basic values of skinfolds and not the results of the evaluation of total fat from skinfolds using our regression equations, derived before for Czech children (7–12) and adolescents (13–18 years) [24]. Using formulas derived for local populations gives always mostly exact results and is therefore recommendable; however none were available for Czech preschool age. The same applies for others [36, 37].

Along with reduced development of lean body mass the insufficient development of bone density can be also considered. Significantly increased adiposity of preschoolers during the periods of our measurements however corresponds to the shift AR to lower age along with the decrease of physical activity levels using pedometers [20] or later by accelerators [6].

Increase of adiposity characterized by skinfolds corresponds to negative changes of motor abilities which can be considered as a marker of physical activity level and resulting energy expenditure. This situation characterizes positive energy balance even under conditions of adhering to recommended dietary allowances (RDA). Children who have been getting fatter more recently became also clumsier as compared to children more decades ago and therefore also less interested in active games and exercise. These have been moreover much less available than in the period when our studies were started.

This finding can be considered not only as a marker of reduced physical fitness, but also as a health risk, which was confirmed also by increased deposition of fat on the trunk where the changes have been mostly marked. This type of fat deposition is considered especially as undesirable, as it characterizes morphologically those who can be more threatened by increased development of metabolic syndrome, which has been revealed more often at younger age than before [22, 23].

Not only increased subcutaneous fat, but also the results of motor tests as markers of the level of physical activity and exercise emphasize the importance of necessary motor stimulation for a desirable development of children with regard not only to physical fitness, but also to health status and its prognosis. Reduced level of motor performance concerning explosive strength of lower extremities (broad jump) and manipulative skill abilities (throwing a ball) was revealed especially in 6-year-old boys, when secular differences in adiposity were also mostly marked and most highly significant and concerned also the changed distribution of fat (trunk skinfolds).

With regard to time period the most marked differences have occurred especially between the last (2012) and before-last measurements (1990), that is, during the period of significant social and economic changes in the Czech Republic. It is necessary to emphasize that obviously due to mentioned changes the lifestyle has changed too, which resulted also in the increase of overweight and obesity prevalence generally in all age categories including children [1, 4, 5].

Increasing prevalence and global epidemy of obesity has concerned more recently also young age categories in many other countries as shown by the comparison of measurements of children in 144 countries [38]. This is also related to the results of the secular increase of adiposity along with the worsening of motor development as a marker of physical activity and energy expenditure level revealed in numerous school age and adolescent populations [12, 13, 35]. De Onis et al. [38] evaluated the changes of height and weight from numerous countries, but not direct characteristics of adiposity, for example, skinfold thickness, which can secularly change without marked changes of BMI [13]. Skinfold thicknesses correlate significantly with total body fat and give more exact information on adiposity as compared with height and weight and their relationship, or BMI only [24, 36, 37]. Other studies followed skinfolds in preschoolers and compared various periods of time but did not assess simultaneously also the parameters of motor and functional development which reflect the level of adaptation to increased and/or decreased physical activity.

Polish authors measured height, weight, and body fat of 1,970 girls and compared the data with the results from previous surveys (1983 and 2000). In all girls measured in 2010, the time of AR was found earlier than in girls measured in 1983 [39]. Authors concluded that earlier AR cannot be explained only by the changes in body adiposity: it could be a marker of acceleration of development which started already in an early postnatal ontogenesis. During last 30 years significant increase of BMI was not revealed; however, an increase of trunk fat was found, especially in boys [40, 41]. One of the causes of these changes could be increasing energy imbalance due to inactivity since earliest periods of life.

As apparent from our study, BMI has not revealed in preschool children similar secular changes as adiposity [42]. This was obviously due to the increase of both height and weight values, which were mostly synchronic and mutually corresponding; only insignificant fluctuations which were similar at any time were revealed. In contrast to that, skinfold measurements differed significantly along time, similarly as the results of motor testing. This finding can be explained by significant secular changes of body composition—increased adiposity due to the reduction of energy expenditure resulting from decreased physical activity level, and reflected also by decreased level of motor performance. In this respect, results of motor testing which characterize worsened functional development and skill of obviously more inactive children correspond to body composition changes—increasing adiposity.

Modifications of the way of life during the mentioned five decades characterized by sedentarism and inadequate food intake as related to energy output influenced negatively both adiposity and motor performance already in preschool children. The most common present nutritional phenomenon is undesirable composition of food (increased intake of saturated fats, simple sugars, etc.) which has especially most undesirable consequences under conditions of reduced energy expenditure due to sedentarism [1, 6, 7]. Most marked changes between years 1990 and 2012 when significant social, economic, cultural and further changes in the Czech Republic occurred were revealed. Moreover, mostly increased deposition of fat on the trunk which is considered as a marker of possible development of metabolic syndrome [22, 23] was apparent already in preschool age, indicating the importance of early intervention concerning also physical activity and availability for exercise since early life. Results concerning AR shift to younger age which correspond to secular changes of skinfold measurements indicate also the increased risk of obesity development in youngest children, which is from the point of view of further increased adiposity the highest. Obesity which develops already in preschoolers has been considered as much greater risk for later increase in obesity prevalence and in this case also accompanied with, for example, metabolic syndrome, psychological, orthopedic, and many other health problems [1, 2].

Epidemiological and research data indicate the essential role of physical activity in energy expenditure, energy balance, and adiposity of the organism. Active individuals are always characterized by lower relative and absolute amount of body fat (provided that their physical activity is of special character—aerobic, dynamic one has adequately higher intensity, duration, and frequency), increased functional capacity, aerobic power, and motor development. This applies even when food intake is increased, as apparent, for example, in athletes and exercising youth who have higher energy intake than recommended dietary allowances. The effect of adequate exercise was revealed in all age categories starting with preschool to advanced age [16, 21, 25, 34] and was therefore included also in complex treatment and prevention of obesity. As emphasized also more recently, longer lasting obesity during growth period also results in musculoskeletal problems (flat feet, back pain, etc.) which further reduce physical activity and worsen the overall situation with regard to proper energy balance. In hypokinetic subjects adiposity increases even when recommended dietary allowances are adhered to.

## 5. Conclusions

Adequate physical activity regime makes possible food intake and behaviour according to individual will and appetite, which is especially recommendable for children. Adhering to special composition of “healthy diets” which includes all indispensable components (RDA) is always a difficult task for any family. So the availability of suitable physical activity regime is the best approach for young children due to their natural trend for highest spontaneous activity in life [4, 5, 20, 35, 43]. “Positive health” can be achieved therefore using physiological means like an adequate physical activity and exercise, which has been however known and experienced long time ago, however, more and more difficult under present conditions of life.

Secular increase of body fat along with the deterioration of motor development was revealed due to sedentarism as early as in preschool age. This can occur even when, for example, average recommended dietary allowances have not been trespassed significantly, and BMI has not been markedly increased, or even not at all. Intervention in physical activity regime should start in the family due to desirable model of parents, siblings, caretakers, and so forth. Spontaneous games in suitable indoor and outdoor areas under the supervision of close caretakers are preferred and should be available as often as possible. Individual approach and individual tailoring according to the characteristics of the child (age, gender, degree of development, environmental conditions, etc.) should be used. Special organized physical education classes together with, for example, one of the parents, or other close caretakers, should be arranged, as positively experienced in more countries. Due to present health complications and their economic consequences in prezent environmental conditions, inteventions in physical activity regimes of children should be guaranteed by health and pedagogic caretakers and also governmental institutions.

Achievement of an increased physical fitness and adequate body composition belongs to the preventive health measures concerning especially the diseases of civilization. As follows from many studies, present inadequate dietary intake which does not correspond to real needs due to reduced energy output and lack of exercise can initiate undesirable overall status of the growing organism already quite early in life especially under conditions of sedentarism.

## Figures and Tables

**Table 1:** Sample characteristics: age, sex—frequency.

Age	sex	Study					
		1957	1977	1980	1985	1990	2012
5	M	166	196	—	—	363	157
	F	157	180	—	—	383	161

6	M	154	156	146	182	233	133
	F	162	178	146	190	198	137

M = male, F = female.

**Table 2:** Sample characteristics: BMI, skinfold thicknesses, and motor abilities.

Survey/year	1957	1977	1980	1985	1990	2012
Anthropometric parameters	+	+	+	+	+	+
Skinfolds	+	+	+	+	+	+
Standing jump	−	+	−	−	−	+
Throw the ball	−	+	−	−	−	+

**Table 3:** Statistical significance of differences between the values of skinfold thicknesses—comparison of the sample followed up in 2012 compared to samples followed up in 1957, 1977, 1980, and 1985.

2012/		1957	1977	1980	1985	1990
Triceps						
Boys	5 years	∗∗∗	∗	—	—	∗
	6 years	∗∗∗	∗∗∗	∗∗∗	∗	∗
Girls	5 years	∗∗∗	∗	—	—	n.s.
	6 years	∗∗∗	n.s.	∗∗∗	n.s.	n.s.
Subscapular						
Boys	5 years	∗∗∗	∗∗∗	—	—	∗∗∗
	6 years	∗∗∗	∗∗∗	∗∗∗	∗∗∗	∗∗∗
Girls	5 years	∗∗∗	∗∗∗	—	—	∗∗∗
	6 years	∗∗∗	∗∗∗	∗∗∗	∗∗∗	∗∗∗
Suprailiac						
Boys	5 years	∗∗∗	∗∗∗	—	∗∗∗	∗∗∗
	6 years	∗∗∗	∗∗∗	∗∗∗	∗	∗∗∗
Girls	5 years	∗∗∗	∗∗∗	—	—	∗∗∗
	6 years	∗∗∗	∗∗∗	∗∗∗	∗∗∗	∗∗∗

Based on three-way ANOVA (age, sex, and year of study); ^∗^ *P* < 0.05; ^∗∗∗^ *P* < 0.001; n.s. = no significance.
